# Combined abiraterone acetate plus prednisone, salvage prostate bed radiotherapy and LH-RH agonists (CARLHA-GEP12) in biochemically-relapsing prostate cancer patients following prostatectomy: A phase I study of the GETUG/GEP

**DOI:** 10.18632/oncotarget.25189

**Published:** 2018-04-24

**Authors:** Stéphane Supiot, Loic Campion, Pascal Pommier, Mélanie Dore, Clément Palpacuer, Séverine Racadot, Emmanuel Rio, Gérard A. Milano, Céline Mahier - Ait Oukhatar, Christian Carrie

**Affiliations:** ^1^ Departments of Radiation Oncology and Biostatistics, Institut de Cancérologie de l'Ouest, Nantes, France; ^2^ Department of Radiation Oncology, Centre Léon Berard, Lyon, France; ^3^ Laboratoire d’Oncopharmacologie, Centre Antoine-Lacassagne, Nice, France; ^4^ Groupe des Essais Précoces, Unicancer, Paris, France

**Keywords:** abiraterone, prostate cancer, prostate bed radiotherapy, salvage radiotherapy, rising PSA

## Abstract

**Background:**

To establish the maximum tolerated dose of abiraterone acetate plus prednisone (AA) combined with salvage radiotherapy (SRT) and goserelin in a phase 1 study in men with rising PSA following radical prostatectomy.

**Methods:**

AA was given during one month before SRT at 1000 mg PO once daily, then 750 mg (Dose Level 1, DL1) or 1000 mg (DL2) during 5 months combined with 6-months goserelin by injection on the first day of irradiation (scheme NEO) or one month before starting SRT (scheme CONCO).

**Results:**

In scheme NEO at DL1, 2/9 patients did not achieve castration levels of testosterone. 4/9 patients (44%) presented with grade 3 liver enzyme elevation. In scheme CONCO testosterone dropped to undetectable levels. At DL1, 6 patients were recruited, with no dose limiting toxicities. At DL2, 2/3 patients presented with grade 3 liver enzyme elevation occurring during SRT.

**Conclusions:**

When AA was administered without goserilin, only 78% achieved castration levels. AA combined with SRT and goserilin did not increase pelvic toxicity, but lead to an unsuspected high frequency of grade 3 liver toxicity. The phase II recommended dose of AA combined to goserelin and SRT is 750 mg.

## INTRODUCTION

Despite adequate prostatectomy, up to 50% of localized prostate cancer patients may relapse biochemically [1]. As soon as the prostate specific antigen (PSA) is confirmed above 0.2 ng/ml, these patients are routinely offered salvage prostate bed radiotherapy, which achieves a biochemical control rate of 60% at 5 years [2]. Two studies showed that adding hormone therapy (HT) to salvage prostate bed radiotherapy was able to significantly increase biochemical relapse-free survival to 80% at 5 years [2] and increase overall survival at 12 years [3]. Combined HT and salvage radiotherapy can now be considered as standard of care in biochemically-relapsing prostate cancer patients.

To increase the systemic efficacy of HT, novel androgen-receptor (AR) signalling targeted agents have been developed [4]. Abiraterone acetate is an orally administered small molecule that irreversibly inhibits CYP17, a rate-limiting enzyme in androgen biosynthesis, and blocks the synthesis of androgens in the testes, adrenal glands and prostate [5]. The abiraterone metabolite Δ(4)-abiraterone is also able to directly block the androgen receptor signalling pathways [6]. Abiraterone acetate plus prednisone is approved for the treatment of metastatic castration resistant [7, 8] and hormone-sensitive [9, 10] prostate cancer.

As there is no prospective data on the combination of abiraterone acetate plus prednisone and salvage prostate bed radiotherapy, the aim of this study was to further evaluate the safety profile of abiraterone acetate plus prednisone in patients with prostate cancer who are biochemically relapsing after surgery and undergo salvage radiotherapy with 6-months LH-RH agonist. We hypothesized that the toxicity profile of both treatments should not potentiate each other.

The CYP17A1 gene presents numerous single nucleotide polymorphisms (SNPs), whose frequencies of rare alleles are at least 12%. Their functional impact has been suggested for nine of them, which were linked either to the risk of developing prostate cancer or to survival of prostate cancer patients [11]. So far, no study has examined the links between these polymorphisms and the effects of a CYP17A1 inhibitor. Also, relationships with the efficacy of androgen deprivation therapy have recently been reported for SNPs of genes involved in the membrane-transport testosterone and dehydroepiandrosterone, namely SLCO2B1 and SLCO1B3 [12–14]. The present study proposed an original approach in order to highlight a relationship between abiraterone acetate plus prednisone activity and patient’s genetic profile.

## RESULTS

### Patient population

Between 12/2012, and 01/2016, 9 and then 9 additional patients were treated in scheme NEO and CONCO respectively (Table 1). 8 out of 9 patients treated in the scheme NEO were evaluable for DLT assessment, while 9/9 were evaluable for DLT in the scheme CONCO. At least one risk factor of relapse following salvage radiohormonotherapy (PSA > 0.5 ng/ml; PDA DT < 7 months; negative surgical margins [2]) was found in most patients (7/9 in scheme NEO and 8/9 in scheme CONCO respectively).

**Table 1:** Patients characteristics

	NEO	CONCO	Total
Number of patients	9	9	18
Median age (range)	57	66	65
Median PSA (ng/ml; range)	0.29 (0.21-0.8)	0.44 (0.26-1.48)	0.43 (0.21-1.48)
pT2	7	5	12
pT3a	0	2	2
pT3b	2	2	4
Positive margins	5	5	10
Gleason score 7 (3+4)	5	6	11
Gleason score 7 (4+3)	4	2	6
Gleason score 9 (4+5)	0	1	1
Median PSA doubling time (months; range)	6 (2-27)	8 (2-13)	7 (2-27)

### Testosterone and LH serum levels

**Figure 1:**
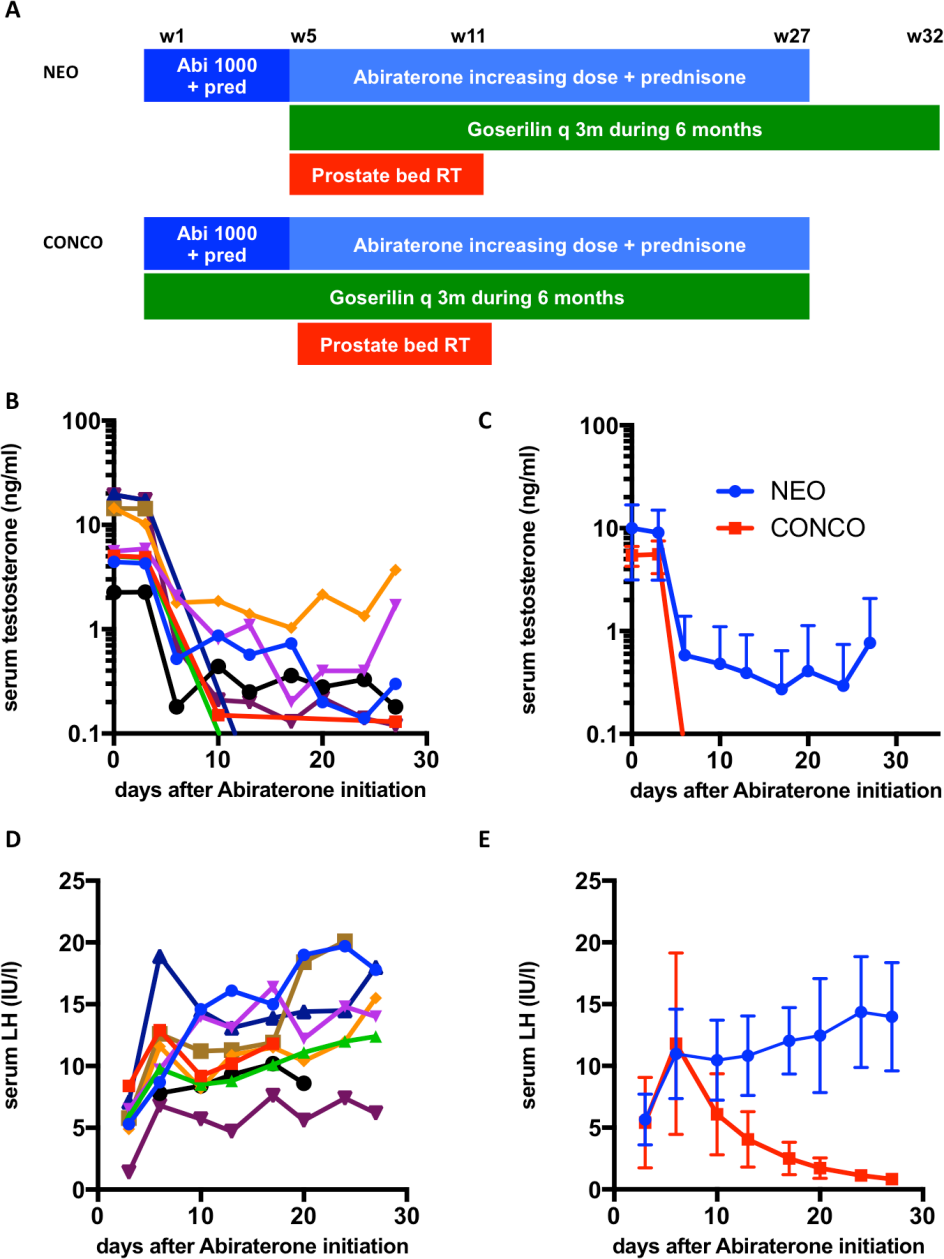
Testosterone and LH levels following different mode of administration of abiraterone **(A)** schematic representation of the NEO and CONCO schemes; **(B)** serum testosterone levels following neo adjuvant (NEO) abiraterone administration in all 9 patients; **(C)** serum LH levels following neo adjuvant (NEO) abiraterone administration in all 9 patients; **(D)** serum testosterone levels following neo adjuvant (NEO) or concurrent (CONCO) abiraterone administration (mean+/- SD); **(E)** serum LH levels following neo adjuvant (NEO) or concurrent (CONCO) abiraterone administration (mean+/- SD).

When abiraterone acetate (1000 mg) plus prednisone was administered without goserelin (scheme NEO), testosterone levels dropped to less than 50 ng/dl (castration levels) after a median time of 10 days (Figure 1B). However, 2/9 (22%) patients did not achieve castration levels of testosterone. After one month, goserelin was added and subsequently all patients achieved undetectable testosterone levels. Median LH levels increased to 10.4 and 12.5 IU/l at day 10 and 20 respectively in all patients (Figure 1C). CYP17A1, SLCO2B1, SLCO2B3, CYP3A4 CYP3A5 polymorphism was determined in all patients (Supplementary Table 1) but did not correlate with a lack of castration on day 27.

When abiraterone acetate plus prednisone was administered with concurrent goserelin (scheme CONCO), testosterone levels dropped to undetectable levels at day 6, while median LH levels decreased to 6.1 and 1.7 IU/l at day 10 and 20 respectively (Figure 1D & 1E). All patients except 3 with a short follow-up (12 months) recovered testosterone levels above castration levels (> 50 ng/dl).

### Toxicity analysis

We started with the NEO scheme at DL1 (Table 2). Abiraterone acetate plus prednisone combined with LH-RH agonists and radiotherapy did not lead to in-field grade 3 bladder or rectum toxicity. One patient was hospitalized with grade 3 renal insufficiency following grade 3 febrile (39.3°C) diarrhea and vomiting that resolved within 2 days with IV antibiotics during the 5^th^ week of radiotherapy (SRT at 52 Gy). CRP was highly elevated (237 mg/ml). Abiraterone acetate plus prednisone was continued during this episode, but radiotherapy was discontinued for 2 days. This toxicity was considered as infectious and unrelated to the treatment.

**Table 2:** Dose levels and dose-limiting toxicities

Scheme	DL	Patients	Abiraterone acetate dose (mg/d)	DL escalating order	DLT	Description
NEO	DL1	9 (1NE^*^)	750	1	3	3 patients with G3 ALT/AST liver enzyme elevation
CONCO	DL1	3 + 3	750	2 4	0	
CONCO	DL2	3	1000	3	2	2 patients with G3 ALT/AST liver enzyme elevation

^*^ Non evaluable for DLT (treatment stopped before DLT evaluation period due to grade 3 ALT/AST liver enzyme elevation).

At dose level 1, 4/9 patients (44% 95% CI [13.7-78.8]) presented with grade 3 liver function tests elevation (ALT and/or AST>5 x ULN), occurring prior to RT or during SRT at 8 Gy, 36 Gy and 54 Gy. The 3 cases of liver toxicities occurred during SRT in 3 out of 6 patients evaluable for DLT were considered as DLT (Table 3). A septic shock following lung infection occurred in a patient with a past history of bronchiectasis 3 months after completion of radiotherapy. This toxicity was considered as unrelated to the treatment. No other grade 3 out-of-field toxicity was reported.

**Table 3:** Adverse events Only the highest grade of toxicity is reported

Scheme	NEO			CONCO			CONCO		
Abi Ac dose during RT	750 mg			750 mg			1000 mg		
Number of patients	9			6			3		
Toxicity grade	1	2	3	1	2	3	1	2	3
**In-field side-effects**									
**Bowel toxicity**									
Diarrhea							1		
Constipation				1			1		
Excessive rectal mucus	1								
Rectal bleeding	2			3					
Rectal urgency	1				1				
Anorectal pain	3			4					
Abdominal pain				1					
*Number of patients with bowel toxicities*	5	0	0	6	1	0	1	0	0
**Bladder toxicity**									
Bladder urgency	3								
Urine incontinence	3	1		2	1	1	1		
Nocturia	1						1		
Pollakiuria	3	1		3			1	1	
Bladder pain				1					
Dysuria	1								
*Number of patients with bladder toxicities*	7	1	0	3	1	1	2	1	0
**Out-of-field side-effects**									
**Liver**									
Increased ALT		1	3	2	1			1	2
Increased AST	1	1	3	3				1	2
Increased bilirubin							2		
*Number of patients with liver toxicities*	1	2	4	3	1	0	2	1	2
**Cardio-vascular**									
Arterial hypertension		4	2	1	5			3	
Bradycardia	1								
Tachycardia	1								
Phlebitis		1							
*Number of patients with cardio-vascular toxicities*	2	4	2	1	5	0	0	3	0
**Other treatment-related toxicities**									
Asthenia	3			2	1		1		
Hot flashes	3	2		4			2		
Headaches	2								
Depression	1	1							
Breast pain	1								
Muscle pain or muscle cramps	2								
Anemia	1								
Hypokalemia		1		1					
Leucopenia	1								
Lymphopenia	1		1	1	4	1	1		
Monopenia	1								
Thrombopenia	1			1					
*Number of patients with other treatment-related toxicities*	6	4	1	5	5	1	2	0	0
**Total number of patients with toxicities**	9	6	5	6	6	2	3	3	2

Adverse events considered not related to treatment were: grade 4 septic shock following lung infection in a patient with bronchiectasis (n=1), grade 3 renal insufficiency in a patient with grade 3 febrile diarrhea (n = 1), grade 2 eczema (n=1), grade 1 skin dryness (n=1), grade 1 mycosis (n=1), grade 1 laryngitis (n=1), grade 1 tinnitus (n=1), grade 1 osteopenia (n=1), grade 2 painful kidney lithiasis (n=1), grade 1 lumbar pain (n=1), grade 1 dyspepsia (n=2), grade 1 conjunctival haemorrhage (n=1).

We hypothesized that this unexpected liver toxicity was related to the lack of concurrent LH-RH agonist. Therefore, we decided to modify LH-RH administration (scheme CONCO) and recruited 9 more patients. Grade 3 urinary incontinence was reported in one patient. No grade 3 rectal toxicity was reported.

At DL1 (750 mg abiraterone acetate during radiotherapy), no grade 3 out-of-field toxicity was reported, nor any DLT, on 3 out of 3 patients. The dose was escalated to DL2, where 2/3 patients presented a DLT with grade 3 liver function tests elevation occurring during SRT at 14 Gy and 24 Gy and the third patient presented a grade 2 liver function tests elevation. To confirm the MTD, up to 6 patients were recruited at DL1. Among these 3 supplementary patients, no liver toxicity, nor any other DLT was reported. We concluded that the MTD of abiraterone acetate + prednisone + LH-RH agonist + salvage pelvic radiotherapy was 750 mg during the course of radiotherapy. CYP17A1, SLCO2B1, SLCO2B3, CYP3A4 CYP3A5 polymorphism was determined in all patients (Supplementary Table 1) but did not correlate with the occurrence of liver toxicity. At DL1, only one grade 3 out-of-field toxicity (lymphopenia) was reported. No grade 3 hypokalemia, cardiac disorders or fluid retention/edema was reported.

### Efficacy

After a median follow-up of 30.6 months (range 12-48), biochemical relapse-free survival was 89% (one patient with a PSA of 0.7 ng/ml at 36 months in the NEO group DL1 without liver toxicity) (Supplementary Figure 2).

## DISCUSSION

Radiotherapy combined with hormone therapy is a standard of care for prostate cancer at many stages [15], but limited information is available regarding the combination of next generation androgen-receptor signaling targeted drugs and radiohormonotherapy [16]. This phase 1 study is the first to analyze the toxicity of abiraterone acetate plus prednisone combined with prostate bed radiotherapy and LH-RH agonists comparing a neoadjuvant and a concurrent scheme of administration of abiraterone acetate plus prednisone. Full dose abiraterone acetate (1000 mg) plus prednisone had never been previously evaluated without LH-RH agonists. This phase 1 study shows that 22% of patients did not achieve castration levels when abiraterone acetate plus prednisone was administered without LH-RH agonists, precluding from the routine use of abiraterone acetate plus prednisone without LH-RH agonists. When combined with prostate bed radiotherapy and LH-RH agonists, abiraterone acetate plus prednisone did not increase bladder or intestine toxicity. Surprisingly, the combined treatment led to an unsuspected high frequency of grade 3 liver toxicity. The P2RD of abiraterone acetate plus prednisone combined to goserelin and SRT was therefore set to 750 mg using a concurrent scheme of administration.

The effects of abiraterone acetate plus prednisone without LH-RH agonists in prostate cancer patients is not well documented. The only phase 1 study that evaluated the effects of abiraterone acetate plus prednisone without LH-RH agonists was limited to a short treatment duration (12 days) and a dose lower (800 mg) than the currently recommended dose (1000 mg) [5]. When abiraterone acetate plus prednisone was administered during one month before starting LH-RH agonists and radiotherapy in 9 patients, only 7/9 (78%) of patients achieved castration levels of testosterone. This lack of castration is probably due to the feedback increased LH levels that LH-RH agonists abrogate. The interpatient variability of abiraterone pharmacokinetics is quite high. The mode of administration of abiraterone acetate was similar in all patients. Abiraterone acetate plus prednisone’s efficacy might be affected by CYP17A1 polymorphism [17, 18], but no specific profile could be determined in the 2 patients who did not achieve castration levels of testosterone. In general, the pharmacogenetics translational study was inconclusive. It covered CYP17A1-linked SNPs and SNPs of genes controlling testosterone and dehydroepiandrosterone transport gene-linked SNPs. The reason for this negative result may be due to the limited number cases of the present study. Altogether, these results suggest that abiraterone acetate plus prednisone should not be administered without LH-RH agonists.

Abiraterone acetate plus prednisone treatment did not increase the pelvic toxicity of radiotherapy. Toxicity of pelvic radiotherapy is usually limited to the bladder or the intestine with a rate of grade 3 acute toxicity of less than 5%. Combined abiraterone acetate plus prednisone, salvage prostate bed radiotherapy and LH-RH agonists did not acutely damage the bladder or the intestine. Only one patient (6.2%) suffered from grade 3 acute bladder incontinence during the course of radiotherapy, which can be related to both previous prostatectomy and radiotherapy. Only one patient suffered from grade 3 diarrhea complicated by renal insufficiency, but it was considered as unrelated to the treatments because the patient was febrile and the diarrhea resolved with IV antibiotics. Abiraterone can induce hypokalemia secondary to hyperaldosteronism and radiotherapy-induced diarrhea could increase this risk. No hypokalemia was reported during the combined therapy. This lack of increased pelvic toxicity is similar to another study in locally advanced cancer where abiraterone acetate plus prednisone was combined with LH-RH agonists and prostate and pelvic lymphnodes radiotherapy [19].

The P2RD of abiraterone acetate plus prednisone combined with prostate bed radiotherapy and LH-RH agonists is 750 mg (DL1) since this combination at dose level 2 lead to an unsuspected high frequency of grade 3 liver toxicity (6/18, 33% 95%CI [13.3-59.0] considering all groups; 2/3, 66% 95%CI [9.4-99.2] considering only patients in the CONCO group DL2). This liver toxicity when combined with radiotherapy is much higher than previous reports on abiraterone acetate plus prednisone without radiotherapy in the localized or metastatic setting where grade 3 liver toxicity was noted in only 3-10% of patients [7, 8, 19, 20]. No direct comparison can be done between large randomized phase 3 studies and our small phase 1 series, and the increased liver toxicity in our phase 1 study may be due to the small numbers of patients recruited. However, our 33.3% (6/18) rate of liver function tests elevation seems much higher than a rate of 3.4% (27/791; bilateral Fisher test, P<10^-6 ;^ Supplementary Table 2). The cause of hepatic injury from abiraterone is unknown (https://livertox.nlm.nih.gov//Abiraterone.htm). Liver toxicity was more frequent at higher doses with 2/3 patients in the DL2 cohort and only 4/15 in the DL1 patients. In our study, no patient-related risk factor (alcohol consumption, viral infection, concurrent medication) or other drug-related parameters (timing of administration, batch number) could explain this toxicity. Liver toxicity of abiraterone acetate plus prednisone may relate to its mechanism of action in inhibition of CYP17. Polymorphism in CYP17 may be involved in determining susceptibility of tamoxifen-induced hepatic steatosis [21]. We could not find a correlation between CYP17 polymorphism and abiraterone acetate plus prednisone-induced liver function tests elevation. Our results are in contradiction with another phase 1 study where abiraterone acetate plus prednisone was combined with LH-RH agonists and prostate and pelvic lymphnodes radiotherapy that showed that only 9% of patients presented grade 3 liver toxicity [19]. The main differences between the 2 studies are (1) the concurrent use of abiraterone acetate plus prednisone and LH-RH agonists, (2) the longer duration (12 weeks) of neoadjuvant abiraterone acetate plus prednisone, (3) the larger radiotherapy fields including prostate and pelvic lymphnodes in Cho et al. None of these differences can easily explain an increased liver toxicity in our study.

To explain this liver toxicity, we can raise two hypotheses. On the one hand, grade 3 liver toxicity affected more patients in the NEO group (4/9, 44%; 95% CI [13.7-78.8]) than in the CONCO group (2/9 22.2%, 95%CI [2.81-60.00] only at dose level 2). From this observation, we can hypothesize that abiraterone pharmacokinetics might be affected by castration. Indeed, castration using LH-RH agonists increases clearance of docetaxel, thereby reducing its toxicity on neutrophils [22]. To test this hypothesis, we will monitor abiraterone pharmacokinetics in the phase 2 study. On the other hand, liver enzymes raised to grade 3 during the course of radiotherapy in 8/9 patients with a grade 3 liver toxicity. We can also hypothesize that systemic effects of radiotherapy might affect the liver toxicity of abiraterone. The release of inflammatory cytokines can mediate radiation toxicity [23]. In mice, pelvic radiotherapy increased TNF-alpha production [24] and TNF-alpha was shown to induce liver injury [25]. In patients, IFN-γ and IL-6 significantly increased during prostate IMRT [26] and Interferon-gamma exacerbates liver damage [27] and high levels of IL-6 are found in patients with liver function tests elevation [28]. To test this hypothesis, cytokines levels will be measured during the phase 2 study.

Although restricted to a limited number of patients and a short follow-up, biochemical relapse-free survival (89%) is encouraging with only one patient with a PSA above 0.10 ng/ml after a median follow-up of 3 years, despite a vast majority of patients presented at least one risk factor of relapse following salvage radiohormonotherapy. Recent phase 3 studies showed that hormone therapy combined with prostate bed radiotherapy could increase biochemical-relapse free [2] or overall survival [3] in patients with a detectable PSA following radical prostatectomy. Since most relapse following prostate bed radiotherapy occur outside the prostate bed [29], major improvements should be achieved by adding systemic therapy such as novel androgen receptor signaling targeted agents. A phase 2 study with combined abiraterone acetate plus prednisone, salvage prostate bed radiotherapy and LH-RH agonists in a larger patient population is ongoing.

## PATIENTS AND METHODS

### Patients

We conducted a multicenter, prospective, open-label phase Ib study evaluating the efficacy and safety of a 6-month abiraterone acetate plus prednisone plus LH-RH agonist combined with prostate-bed radiotherapy in the Radiotherapy Departments of the Institut de Cancérologie de l’Ouest, Nantes, and Centre Léon Bérard, Lyon, both in France.

Eligible patients were men aged 18 years or older, with histologically confirmed adenocarcinoma of the prostate, stage pT2, pT3, and pT4a (bladder neck involvement only), and pN0, who had received radical prostatectomy. Eligible patients had PSA concentrations of less than 0.1 μg/L for at least 6 months after surgery, which then began to rise (to between 0.2 μg/L and <2 μg/L, as confirmed by two consecutive tests) without evidence of clinical disease (total bone scan and pelvic CT scan). Other inclusion criteria were a performance status of 0–1, serum potassium ≥ 3.5 mmol/l in the 72 hours before first dose of abiraterone, serum creatinine < 1.5 x ULN or a calculated creatinine clearance ≥ 60 mL/min, serum bilirubin < 1.5 x ULN (except for patients with documented Gilbert’s disease), AST and/or ALT < 2.5 x ULN.

Patients were excluded if: they had undergone previous androgen deprivation therapy or pelvic radiotherapy, the initial status at the time of surgery was pN1, histology findings showed cancer other than adenocarcinoma, the patient had another invasive cancer in the previous 5 years, another antineoplastic treatment was in progress, previous hormone therapy including prior therapy with ketoconazole or a CYP17 inhibitor(s) for prostate cancer had been administered, and in case of uncontrolled hypertension (defined as systolic BP ≥ 140 mmHg or diastolic BP ≥ 90 mmHg). Patients with a history of hypertension were allowed provided blood pressure was controlled by anti-hypertensive therapy. Patients with active or symptomatic viral hepatitis or chronic liver disease, severe and moderate hepatic impairment (Child-Pugh class B and C), and patients being treated within the last 14 days prior to inclusion with drugs recognized as being strong inhibitors or inducers of the isoenzyme CYP3A4 (clarithromycin, ketoconazole, itraconazole, voriconazole, ritanovir,) or requiring those treatments during the study were also excluded. Patients with known hypersensitivity to any of the study drugs or excipients, galactosemia, glucose-galactose malabsorption or lactase deficiency, with severe and/or uncontrolled medical disease which could compromise participation in the study, such as, but not limited to clinically significant heart disease as evidenced by myocardial infarction, or arterial thrombotic events in the past 6 months, severe or unstable angina, or New York Heart Association (NYHA) Class III or IV heart disease or cardiac ejection fraction measurement of < 50% at baseline were also excluded.

### Objectives

The objective of this phase 1 study was to determine the maximum tolerated dose (MTD) and the phase II recommended dose (P2RD) of abiraterone acetate plus prednisone combined with radiotherapy and LH-RH agonist treatment in patients with biochemical relapse from prostate cancer following surgery. Exploratory objectives aimed to evaluate potential biomarkers of abiraterone acetate plus prednisone treatment toxicity, and to perform testosterone and LH serum levels assays.

### Toxicity assessment

Toxicities were assessed weekly during the DLT evaluation period and at every visit through a clinical assessment, laboratory examinations and an ECG every 3 months. The DLT evaluation period covered 11 weeks starting from the radiotherapy initiation. Toxicities were scored according to National Cancer Institute (NCI) Common Terminology Criteria for Adverse Events (CTCAE), version 4.0.

### Radiotherapy

All patients underwent radiotherapy starting on day 30 after abiraterone acetate plus prednisone initiation. A total dose of 66 Gy in 33 fractions (5 a week) was delivered to the prostate bed using Intensity Modulated Radiation Therapy. Clinical target volume (CTV) limits were based on the guidelines of the RTOG consensus guidelines [30]. Planning target volume (PTV) was obtained by adjunction of a 7-mm margin in all directions. The following limits were set: less than 50% and 100% of the rectal wall was to receive 60 Gy or 50 Gy respectively; less than 100% of the bladder wall was to receive 50 Gy.

### Abiraterone acetate plus prednisone and LH-RH agonist

During one month before initiating radiotherapy, abiraterone acetate was taken orally at the dose of 1000 mg/day (i.e. four tablets of 250 mg once daily) plus prednisone 5 mg twice daily. Then, abiraterone acetate and prednisone were administered in accordance to the dose-escalation scheme (see below).

HT consisted of a 3-month depot preparation of goserelin administered subcutaneously in two injections separated by a three-month interval. Goserelin was initiated either with radiotherapy (scheme NEO), or concomitantly with abiraterone one month before starting radiotherapy (scheme CONCO) (Figure 1A).

### Dose-escalation scheme

This dose range finding phase I study investigated the maximum tolerated dose (MTD) of abiraterone acetate plus prednisone in combination with prostate-bed radiotherapy and LH-RH agonist, based on dose-limiting toxicities (DLT) occurrence using a 3x3 algorithm model (Supplementary Figure 1). After initiating radiotherapy, abiraterone acetate plus prednisone 5 mg twice daily were taken orally according to a dose seeking procedure starting at 750 mg/day (i.e., 3 tablets of abiraterone acetate 250 mg once daily, DL1). If no DLT was observed during the 11-week DLT evaluation period in the cohort of 3 patients, the next level was open (1000 mg; dose level 2). If one DLT out of three patients (1 DLT/3) was observed, that dose level was expanded to 6 patients. If two DLT, or more, were observed (i.e. ≥ 2 DLTs / 3 or 2 DLTs / 6), this dose level was considered to have exceeded the MTD and the preceding dose level was expanded to 6 patients. Once the MTD was reached, it needed to be confirmed on a total of 6 patients. In case of non-pelvic DLT, abiraterone acetate was definitely withheld and corticoids gradually tapered. Any patient withdrawn before starting radiotherapy or during the DLT evaluation period for any other reason than a DLT was replaced by another patient.

### Pharmacogenomics

We assessed testosterone and LH serum level variations weekly during one month after abiraterone acetate plus prednisone initiation in scheme NEO and CONCO. We assessed 15 single-nucleotide polymorphisms (SNP) on 5 genes. CYP17A1 gene (allele frequency of the rare alleles > 12%) potentially related to the pharmacogenomics of abiraterone (-34 T>C (rs743572), -362 T>C (rs2486758), 35 T>C (rs1004467), 137 G>A (rs6162), 195 C>A (rs6163), 11994 C>A (rs4919683), 13871 A>G (rs10883782), 15831 G>T (rs619824) and 1243+113 T>A (rs10883783)), CSLCO2B1 gene (rs1077858, rs12422149, rs1789693), SLCO1B3 gene (rs4149117), CYP3A4 gene (rs2740574) and CYP3A5 gene (rs776746) by Mass Array (iPlex chemistry from AGENA, San Diego, USA). DNA was extracted from blood on a Maxwell16 IVD extractor (purification kit AS1290, Promega, Wisconsin, USA). DNA was then analyzed by Mass Array. The first step of this analysis corresponds to an amplification of the region around the SNPs of interest, followed by a SAP treatment prior the final extend primer steps. The extension products were then dispensed onto a SpectroCHIP^®*^ array with a nanodispenser RS100 (AGENA) and detected via MassARRAY MALDI-TOF mass spectrometry (MassARRAY Dx Analyzer 4, AGENA) and the mass spectra analyzed by the TyperAnalyzer software (4.0.26.75).”

### Statistical analysis

The primary objective of this phase I study was to determine the MTD of abiraterone acetate plus prednisone combined with radiotherapy and LHRH agonist treatment in patients with biochemical relapse from prostate cancer following surgery. A dose-escalation scheme based on a 3+3 algorithm model was planned (Supplementary Figure 1). The MTD was defined as the highest of the dose level associated with strictly less than 2 out of 3 or 6 patients presenting with a DLT. A DLT was defined as any treatment-related toxicity occurring over a 11-week period starting from radiotherapy initiation and ending 4 weeks after the radiotherapy termination. Toxicities occurring during the abiraterone acetate plus prednisone pretreatment period of 30 days were not considered as DLTs but were reported as adverse events. Toxicities were graded according to the National Cancer Institute Common Terminology Criteria (NCI CTC, version 4.0).

All patients who received any study agent were monitored for toxicity and included in the safety analysis. To be evaluable for DLT, a patient must either be observed for a full DLT evaluation period, (i.e. 11 weeks from the radiotherapy initiation) or have experienced a DLT.

Statistical analyses were performed with SAS statistical software, V9.4 (SAS Institute, Cary, NC, USA).

## CONCLUSIONS

This study is the first to show that full dose abiraterone acetate (1000 mg) plus prednisone (5 mg BID) without LH-RH agonists is not able to achieve a durable testosterone decrease at castration levels, due to LH feedback increase. The use of abiraterone acetate plus prednisone without LH-RH agonists is therefore not recommended. Moreover, this phase 1 study is the first to show that abiraterone acetate plus prednisone combined with prostate bed radiotherapy and LH-RH agonists does not increase bladder or intestine toxicity, but surprisingly leads to a frequent grade 3 ALT/AST liver enzyme elevation. The recommended dose of concurrent abiraterone acetate plus prednisone (5 mg BID), LH-RH agonists and prostate bed radiotherapy is 750 mg.

## SUPPLEMENTARY MATERIALS FIGURES AND TABLES


