# Supplementary material for: Combined abiraterone acetate plus prednisone, salvage prostate bed radiotherapy and LH-RH agonists (CARLHA-GEP12) in biochemically-relapsing prostate cancer patients following prostatectomy: A phase I study of the GETUG/GEP
Source: Oncotarget. 2018 Apr 24;9(31):22147–57. doi: 10.18632/oncotarget.25189 (PMC5955159; doi:10.18632/oncotarget.25189)
Supplement: Supplementary file 1 [file oncotarget-09-22147-s001.pdf]

Combined abiraterone acetate plus prednisone, salvage prostate bed radiotherapy and LH-RH agonists (CARLHA-GEP12) in biochemically-relapsing prostate cancer patients following prostatectomy: A phase I study of the GETUG/GEP

SUPPLEMENTARY MATERIALS

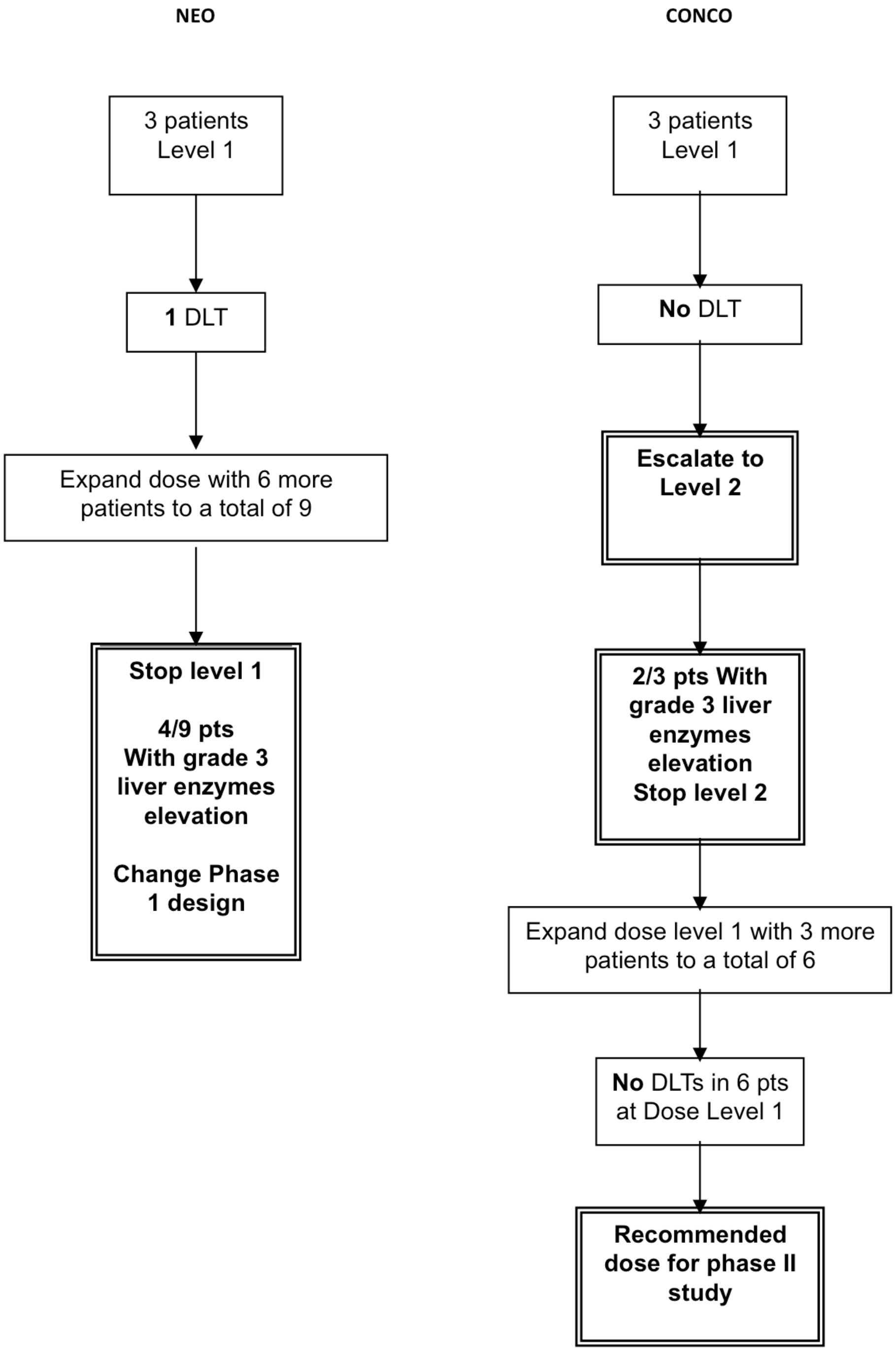

Supplementary Figure 1: Dose-seeking procedure.

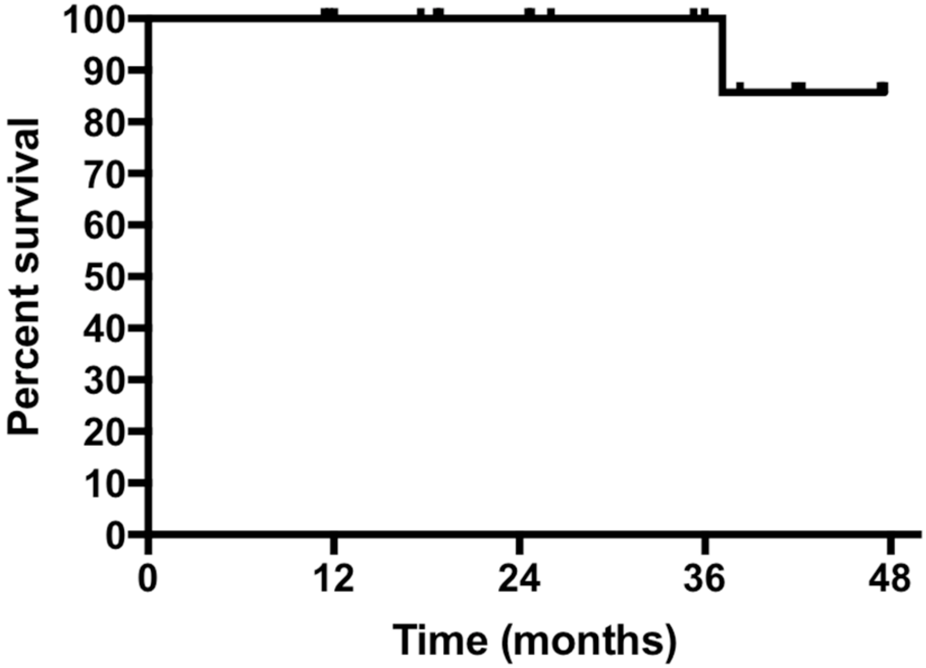

Supplementary Figure 2: Biochemical relapse-free survival.

Supplementary Table 1: CYP17A1, SLCO2B1, SLCO2B3, CYP3A4 CYP3A5 polymorphism nd: not determined

**Patient**			**01**	**02**	**03**	**04**	**05**	**06**	**07**	**08**	**09**	**10**	**11**	**12**	**13**	**14**	**15**	**16**	**17**	**18**	**p-Fisher**
**Grade 3 liver enzyme elevation**			no	yes	yes	no	no	no	yes	yes	no	no	no	no	no	yes	yes	no	no	no	
	SNP ID	**HGVS Name**																			
**CYP17A1**	**rs1004467**	**c.666+35T>C**	TT	TT	TT	TT	TT	TT	TT	TC	TT	TT	TT	TT	TT	TT	TT	TC	TT	TT	1.0
	**rs10883782**	**g.102824175A>G**	AA	AG	AA	AA	AA	AA	AA	AA	AA	AG	AA	AA	AG	AA	AA	AA	AA	AA	1.0
	**rs2486758**	**c.-362T>C**	TT	TT	CC	TT	TT	TT	TT	TT	TT	TT	TT	TT	TT	TT	TC	TT	TT	TT	0.09
	**rs6163**	**c.195C>A**	CA	CA	CC	CC	AA	CC	AA	AA	CA	CA	CC	CA	CA	CC	CC	CA	CA	AA	0.24
	**rs619824**	**g.104581288G>T**	TT	GT	GG	GG	TT	GG	GT	TT	GT	GT	GG	GG	GT	GG	GT	GT	GT	TT	1.0
	**rs743572**	**c.-34A>G**	AG	AG	AA	AA	GG	AA	GG	GG	AG	AG	AA	AG	AG	AA	AA	AG	AG	GG	0.24
	**rs10883783**	**c.1243+113T>A**	TA	TA	TT	TT	AA	TT	AA	TA	TA	TA	TT	TA	TA	TT	TT	TT	TA	AA	0.81
	**rs6162**	**c.138G>A**	AA	GA	GG	GG	AA	GG	AA	AA	GA	GA	GG	GA	GA	GG	GA	GA	GA	AA	1.0
	**rs4919683**	**g.104585125C>A**	AA	CA	CC	CC	AA	CC	AA	AA	CA	CA	CC	CA	CA	CC	CA	CA	CA	AA	1.0
**SLCO2B1**	**rs12422149**	**c.935G>A**	GG	GG	GG	GG	GG	GG	GG	GG	GA	GG	GG	GG	GG	GA	GG	GG	GG	GG	1.0
	**rs1789693**	**c.972+3551T>A**	TT	TA	TA	TA	TA	AA	TT	TT	TT	TT	TA	AA	TT	TA	TT	TT	TA	TT	0,81
	**rs1077858**	**c.1075+2318A>G**	AA	AG	AG	AG	AG	AA	AA	AA	AA	AA	AA	AG	AA	GG	AG	AA	AG	AG	0.29
**SLCO2B3**	**rs4149117**	**c.334G>T**	GT	GG	GG	GG	GG	GT	GG	GG	GG	GG	GG	GG	GG	GT	ND	GT	GT	GG	1.0
**CYP3A4**	**rs2740574**	**c.-392A>G**	AA	AA	AA	AA	AA	AA	AA	AA	AA	AA	AA	AA	AA	AA	AA	AG	AA	AA	1.0
**CYP3A5**	**rs776746**	**c.189-237G>A**	GG	GG	GG	GG	GG	GG	GG	GG	GG	GG	GG	GG	GG	GG	GG	AA	GG	GG	1.0

Supplementary Table 2: Comparison of liver enzyme elevation in different studies using abiraterone

	NEO	CONCO	Total	Cho E, et al.	Taplin ME, et al.	Fizazi K, et al	James ND, et al	de Bono, J. S. et al.	Ryan, C. J. *et al.*
Disease state	Biochemical recurrence following prostatectomy undergoing radiotherapy			Localized intermediate- and high-risk prostate cancer undergoing radiotherapy	Localized high-Risk prostate cancer undergoing prostatectomy	Metastatic, castration-sensitive prostate cancer	Metastatic, castration-sensitive prostate pancer	Metastatic prostate cancer with previous chemotherapy	Metastatic prostate cancer without previous chemotherapy
Grade 3-4 ALT increase	3/9 33.3% 95%CI [7.49%-70.1%]	2/9 22.2% 95%CI [2.81%-60.00%]	5/18 27.8% 95%CI [9.69%-53.48%]		0	33/597 5.6% 95%CI [3.8% - 7.7%]	53/948 5.6% 95%CI [4.2% - 7.2%]		29/542 5% 95%CI [3.61% - 7.59%]
Grade 3-4 AST increase	3/9 33.3% 95%CI [7.49%-70.1%]	2/9 22.2% 95%CI [2.81%-60.00%]	5/18 27.8% 95%CI [9.69%-53.48%]		5/58 8.6% 95%CI [2.86% - 18.98%]	26/597 4.4% 95%CI [2.9% - 6.3%]	10/948 1.1% 95%CI [0.5% - 1.9%]		16/542 3% 95%CI [1.70% - 4.75%]
Grade 3 liver function tests elevation	4/9 44.4% 95%CI [13.7%-78.8%]	2/9 22.2% 95%CI [2.81%-60.00%]	6/18 33.3% 95%CI [9.4% - 99.2%]	2/22 9.1% 95%CI [1.12%-29.16%]	5/58 8.6% 95%CI [2.86 - 18.98]			27/791 3.4% 95%CI [2.26% -4.93%]	
